# Differences in the awareness of stroke symptoms and emergency response by occupation in the Korean general population

**DOI:** 10.1371/journal.pone.0218608

**Published:** 2019-06-18

**Authors:** Gyung-Jae Oh, Kyungsuk Lee, Kyungsu Kim, Young-Hoon Lee

**Affiliations:** 1 Department of Preventive Medicine and Institute of Wonkwang Medical Science, Wonkwang University School of Medicine, Iksan, Jeonbuk, Republic of Korea; 2 Regional Cardiocerebrovascular Center, Wonkwang University Hospital, Iksan, Jeonbuk, Republic of Korea; 3 National Institute of Agricultural Sciences, Rural Development Administration, Jeonju, Jeonbuk, Republic of Korea; Cleveland Clinic, UNITED STATES

## Abstract

We evaluated the difference in awareness of stroke warning signs (SWS) and emergency response among occupational groups in the community-dwelling population. From the 2016 Korea Community Health Survey, a total of 10,445 individuals without stroke were included in the analysis. Multiple logistic regression analysis was used to explore the association of occupation with awareness of SWS and correct emergency response. SWS included the following: sudden numbness or weakness, sudden difficulty speaking or understanding speech, sudden dizziness, sudden visual impairment, and sudden severe headache. Respondents’ occupation was classified into six groups: managers and professionals (MP); clerks; service and sales workers (SSW); agricultural, forestry, and fishery workers (AFFW); mechanical and manual laborers (MML); or housewives and unemployed people (HUP). Awareness of each SWS was the same with the highest for MP and lowest for AFFW. After adjusting for socio-demographic factors, compared to MP (reference), AFFW (odds ratio 0.49; 95% confidence interval 0.36–0.67), HUP (0.55; 0.40–0.75), MML (0.57; 0.42–0.79), and SSW (0.62; 0.45–0.86) had significantly lower ORs for knowing at least one of the SWS. Additionally, AFFW (0.79; 0.66–0.96) and MML (0.76; 0.63–0.91) had significantly lower ORs for knowing all five SWS compared to MP. However, there was no significant occupational difference in correct emergency response when a stroke occurred. To improve stroke literacy and to reduce the disparity of awareness of SWS in community settings, public health efforts with an emphasis on AFFW and MML are needed.

## Introduction

Stroke, one of the most important public health problems in the world, is a disease that can lead to severe disability or death. As early intervention such as thrombolytic therapy is used to treat acute stroke, prompt treatment affects the survival and prognoses of stroke patients [1,2]. However, due to lack of knowledge of stroke and inadequate emergency response, many stroke patients are not getting to the hospital within the golden time. Although not always, many studies have reported that stroke awareness affects early arrival at the hospital [3–5]. Low stroke awareness limits acute stroke care in high-risk populations and makes effective early treatment difficult [6].

To reduce the disparity in stroke knowledge by identifying vulnerable groups with low stroke awareness, it is important to investigate factors related to stroke awareness. Previous studies have reported that socio-demographic factors are significantly related to stroke awareness in the population. Gender [7–11], race [11–13], and age [7–9,13–15] were independently associated with knowledge of stroke warning signs (SWS). Socio-economic factors such as education [7–9,13–17] and income [9,16,18] were also related to stroke awareness. Among the relating factors, older age, ethnic minority, and lower education levels had consistently poor levels of stroke awareness across studies.

Occupation is a good indicator of socioeconomic inequality in health, because it reflects overall income, education, social position, and exposure to hazards [19,20]. Previous studies have shown that occupation is an important risk factor for stroke, by demonstrating differences in stroke morbidity and mortality according to occupation [21–23]. However, to date, little information is available on the differences in stroke awareness based on occupation. Because people spend a lot of time at work, if certain occupational groups are found to have low awareness of stroke, public health interventions targeting these occupations may be effective in reducing the disparity of awareness of stroke in community settings. Therefore, among the community-dwelling population in Korea, we assessed the difference in awareness of SWS and correct emergency response among the occupational groups.

## Methods

### Study population

We analyzed data of the Jeonbuk metropolitan area from the 2016 Korea Community Health Survey (KCHS) conducted by the Korea Centers for Disease Control and Prevention. The KCHS is a nationwide survey conducted by trained surveyors who performed personal interviews. The 2016 KCHS conducted in the Jeonbuk metropolitan area included a total of 12,428 individuals ≥19 years of age. After excluding participants with non-response or refusal to provide sociodemographic data and/or SWS information, 10,445 individuals who had not been diagnosed with stroke were included in the final analysis. Written informed consent was obtained from all participants of the KCHS. The study protocol was approved by the institutional review board of Wonkwang University Hospital [WKUH 2019-02-007].

### Measures

Socio-demographic variables were explored using a standardized questionnaire. Respondents’ age was classified into the following groups: 19–29, 30–39, 40–49, 50–59, 60–69, and ≥70 years. The educational level (non-formal education, elementary school, middle school, high school, or college or higher), monthly household income (<100, 100–199, 200–299, 300–399, 400–499, or ≥500 ten thousand KRW), residence type (rural or urban), and marital status (married and living with a spouse, divorced or separated, widowed, or never married) were also grouped for analysis. We have reclassified the respondent’s occupation into six occupational groups that represent a similar socioeconomic status using the 11 occupations (excluding students and soldiers) collected according to the Korean standard occupational classification: (1) managers and professionals [MP] (①managers, ②professionals and related workers), (2) clerks [CL] (③office workers), (3) service and sales workers [SSW] (④service workers, ⑤sales workers), (4) agricultural, forestry, and fishery workers [AFFW] (⑥agriculture, forestry and fishery workers), (5) mechanical and manual laborers [MML] (⑦functional and related workers, ⑧equipment, machine operation and assembly workers, ⑨simple laborers), (6) housewives and unemployed people [HUP] (⑩housewives and ⑪unemployed). Smoking status was categorized into never, former, and current. Average amounts drank per drinking day were categorized into none, 1–2 drinks, 3–6 drinks, and ≥7 drinks. Diagnoses of hypertension and diabetes were also evaluated.

Respondents were asked the following closed-ended questions to evaluate the awareness of SWS: “If you think the following is a symptom of stroke, please answer ‘Yes’ or if you do not think so, answer ‘No’. If you are unsure, answer ‘I do not know’.” SWS included the following: sudden numbness or weakness (S1), sudden difficulty speaking or understanding speech (S2), sudden dizziness (S3), sudden visual impairment (S4), and sudden severe headache (S5) [5]. Only those who answered ‘Yes’ to question were judged to be aware of the respective stroke symptom. Respondents were also asked, “What do you think you should do first if someone has SWS?” Respondents chose one of the following answers: ‘take them to a hospital,’ ‘take them to an oriental medicine hospital,’ ‘call an ambulance (119),’ ‘contact family,’ ‘do something else,’ or ‘do not know.’ Only those who answered ‘call an ambulance (119)’ were judged to be aware of the correct emergency response.

### Statistical analysis

Categorical variables are expressed as frequencies (with percentages) and continuous variables as means ± standard deviations. Multiple logistic regression analysis was used to explore the association between occupation and awareness of stroke (SWS and emergency response). Three sequential models were employed. Model 1 was unadjusted; Model 2 was adjusted for gender and age; and Model 3 was further adjusted for residence type, marital status, education level, and monthly household income in addition to those in Model 2. Awareness of SWS was assessed as individual knowledge of SWS, knowing at least one of the SWS, and knowing all five SWS. Compared to MP, the odds ratios (ORs) with 95% confidence intervals (CIs) of other occupations were calculated. All statistical analyses were performed using SPSS version 22.0 (IBM Corp., Armonk, NY, USA). *P*-value <0.05 was considered to indicate statistical significance.

## Results

### Characteristics of the participants

Table 1 shows the characteristics of the study population. The mean age of the participants was 58.1±16.4 years and the proportion of females was 57.0%. The occupation distribution of MP, CL, SSW, AFFW, MML, and HUP was 7.5%, 5.8%, 11.7%, 23.4%, 15.4%, and 36.2%, respectively.

**Table 1:** Characteristics of the study population (N = 10,445).

	n	(%)
Gender		
Male	4,491	(43.0)
Female	5,954	(57.0)
Age, years^a^	58.1	± 16.4
Age group, years		
19–29	516	(4.9)
30–39	1,080	(10.3)
40–49	1,673	(16.0)
50–59	2,064	(19.8)
60–69	2,141	(20.5)
≥70	2,971	(28.5)
Residence type		
Rural (eup∙myeon)	7,466	(71.5)
Urban (dong)	2,979	(28.5)
Education level		
Non-formal education	2,069	(19.8)
Elementary school	2,233	(21.4)
Middle school	1,186	(11.3)
High school	2,725	(26.1)
College or higher	2,232	(21.4)
Marital status		
Married and living with a spouse	7,438	(71.2)
Divorced or separated	455	(4.3)
Widowed	1,700	(16.3)
Never married	852	(8.2)
Monthly household income, ten thousand KRW		
<100	3,514	(33.6)
100–199	2,160	(20.7)
200–299	1,895	(18.1)
300–399	1,157	(11.1)
400–499	769	(7.4)
≥500	950	(9.1)
Occupation		
Managers and professionals	782	(7.5)
Clerks	610	(5.8)
Service and sales workers	1,226	(11.7)
Agricultural, forestry, and fishery workers	2,443	(23.4)
Mechanical and manual laborers	1,603	(15.4)
Housewives and unemployed people	3,781	(36.2)
Smoking status		
Never	6,796	(65.0)
Former	1,959	(18.8)
Current	1,690	(16.2)
Average amount drank per drinking day		
None	4,351	(41.7)
1–2 drinks	2,538	(24.3)
3–6 drinks	2,105	(20.1)
≥7 drinks	1,451	(13.9)
Diagnosed with hypertension		
No	7,184	(68.8)
Yes	3,261	(31.2)
Diagnosed with diabetes		
No	9,237	(88.4)
Yes	1,208	(11.6)

^a^Data are presented as number (percentage) or mean ± standard deviation.

Table 2 shows the characteristics of the study population according to occupation. The proportion of males was highest among MML (64.9%) and the proportion of females was highest among HUP (72.4%). Among the six occupation groups, HUP (65.3±17.0 years) was oldest, while CL (42.1±9.3 years) was youngest. The proportion of current smoking was highest among MML and lowest among HUP. The proportions of diagnoses of hypertension and diabetes were highest among HUP and lowest among MP and CL.

**Table 2:** Socio-demographic characteristics of the study population by occupation.

	Managers and professionals (n = 782)		Clerks (n = 610)		Service and sales workers (n = 1,226)		Agricultural, forestry, and fishery workers (n = 2,443)		Mechanical and manual laborers (n = 1,603)		Housewives and unemployed people (n = 3,781)	
Gender												
Male	46.5		47.9		34.7		54.2		64.9		27.6	
Female	53.5		52.1		65.3		45.8		35.1		72.4	
Age, years^a^	43.1	±10.9	42.1	±9.3	50.1	±12.8	63.3	±11.7	52.7	±14.0	65.3	±17.0
Age group, years												
19–29	12.7		9.5		6.9		1.0		5.5		4.3	
30–39	24.8		30.7		13.1		2.8		13.1		6.9	
40–49	33.5		38.0		25.2		8.5		22.8		7.8	
50–59	22.9		18.9		33.0		21.0		27.9		10.7	
60–69	5.4		2.6		16.0		34.9		16.9		20.2	
≥70	0.8		0.3		5.8		31.8		13.8		50.1	
Residence type												
Rural (eup∙myeon)	49.0		52.1		54.6		96.0		62.0		72.9	
Urban (dong)	51.0		47.9		45.4		4.0		38.0		27.1	
Education level												
Non-formal education	0.6		0.0		2.8		22.9		10.8		34.3	
Elementary school	0.9		0.8		14.9		35.2		18.7		23.2	
Middle school	2.3		1.1		13.5		15.5		13.1		10.7	
High school	22.1		27.5		43.0		19.0		41.1		19.4	
College or higher	74.0		70.5		25.8		7.3		16.3		12.3	
Marital status												
Married and living with a spouse	76.7		78.4		75.8		82.8		70.2		60.3	
Divorced or separated	2.9		4.1		7.5		2.9		6.6		3.6	
Widowed	0.9		1.0		6.7		10.8		11.8		30.4	
Never married	19.4		16.6		10.0		3.4		11.4		5.6	
Monthly household income, ten thousand KRW												
<100	3.2		1.8		11.7		40.1		21.6		53.1	
100–199	14.1		9.0		22.6		26.7		22.2		18.8	
200–299	22.5		22.1		25.4		16.6		23.3		13.1	
300–399	18.4		21.0		18.2		6.0		16.3		6.7	
400–499	17.6		21.0		10.0		3.8		8.5		4.0	
≥500	24.2		25.1		12.0		6.9		8.0		4.3	
Smoking status												
Never	66.6		65.4		70.3		57.7		48.4		74.8	
Former	15.2		14.9		12.6		25.7		20.8		16.8	
Current	18.2		19.7		17.1		16.7		30.8		8.4	
Average amount drank per drinking day												
None	24.3		15.9		30.6		40.8		28.3		59.2	
1–2 drinks	28.8		26.7		25.1		27.9		19.3		22.5	
3–6 drinks	25.4		31.8		25.9		19.6		27.6		12.5	
≥7 drinks	21.5		25.6		18.4		11.7		24.9		5.8	
Diagnosed with hypertension												
No	90.4		88.2		79.6		64.1		74.7		58.2	
Yes	9.6		11.8		20.4		35.9		25.3		41.8	
Diagnosed with diabetes												
No	95.7		95.9		91.9		86.8		91.1		84.5	
Yes	4.3		4.1		8.1		13.2		8.9		15.5	

^a^Data are presented as percentage or mean ± standard deviation.

### Awareness of SWS by occupation

**Fig 1:**
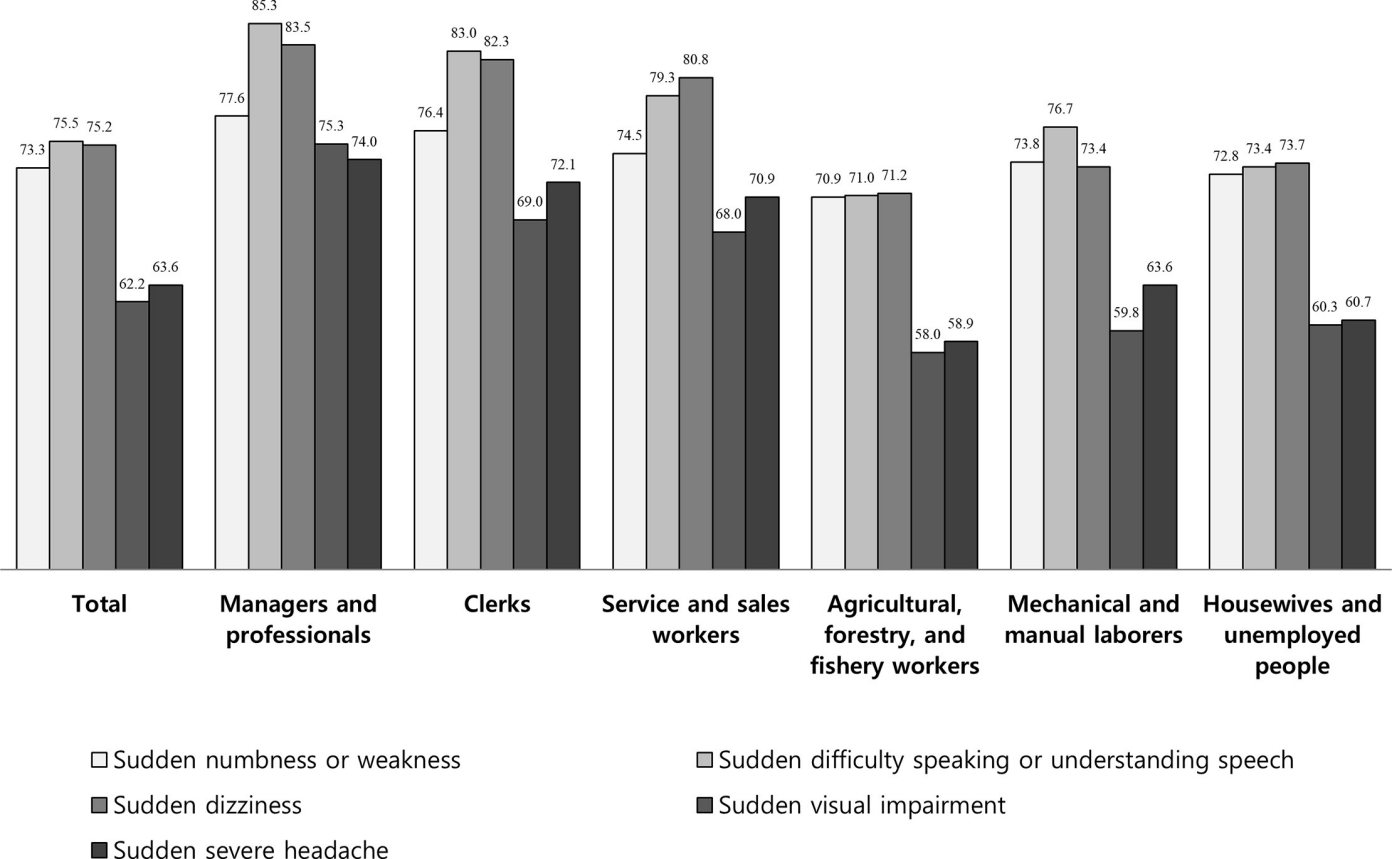
Awareness of individual stroke warning symptoms by occupation. The proportions of the respondents aware of each stroke symptom were relatively higher for S2 (75.5%), S3 (75.2%), and S1 (73.3%), while relatively lower for S5 (63.6%) and S4 (62.2%).

Among all participants, among the five SWS, the proportions of the respondents aware of each stroke symptom were relatively higher for S2 (75.5%), S3 (75.2%), and S1 (73.3%), while relatively lower for S5 (63.6%) and S4 (62.2%). According to occupation, there were differences between stroke symptoms with highest awareness and stroke symptoms with the lowest awareness: MP (S2, the highest vs S5, the lowest), CL (S2 vs S4), SSW (S3 vs S4), AFFW (S3 vs S4), MML (S2 vs S4), and HUP (S3 vs S4). Meanwhile, in all five SWS, the proportions of the respondents aware of each stroke symptom were the same with the highest among MP and lowest among AFFW (Fig 1).

**Fig 2:**
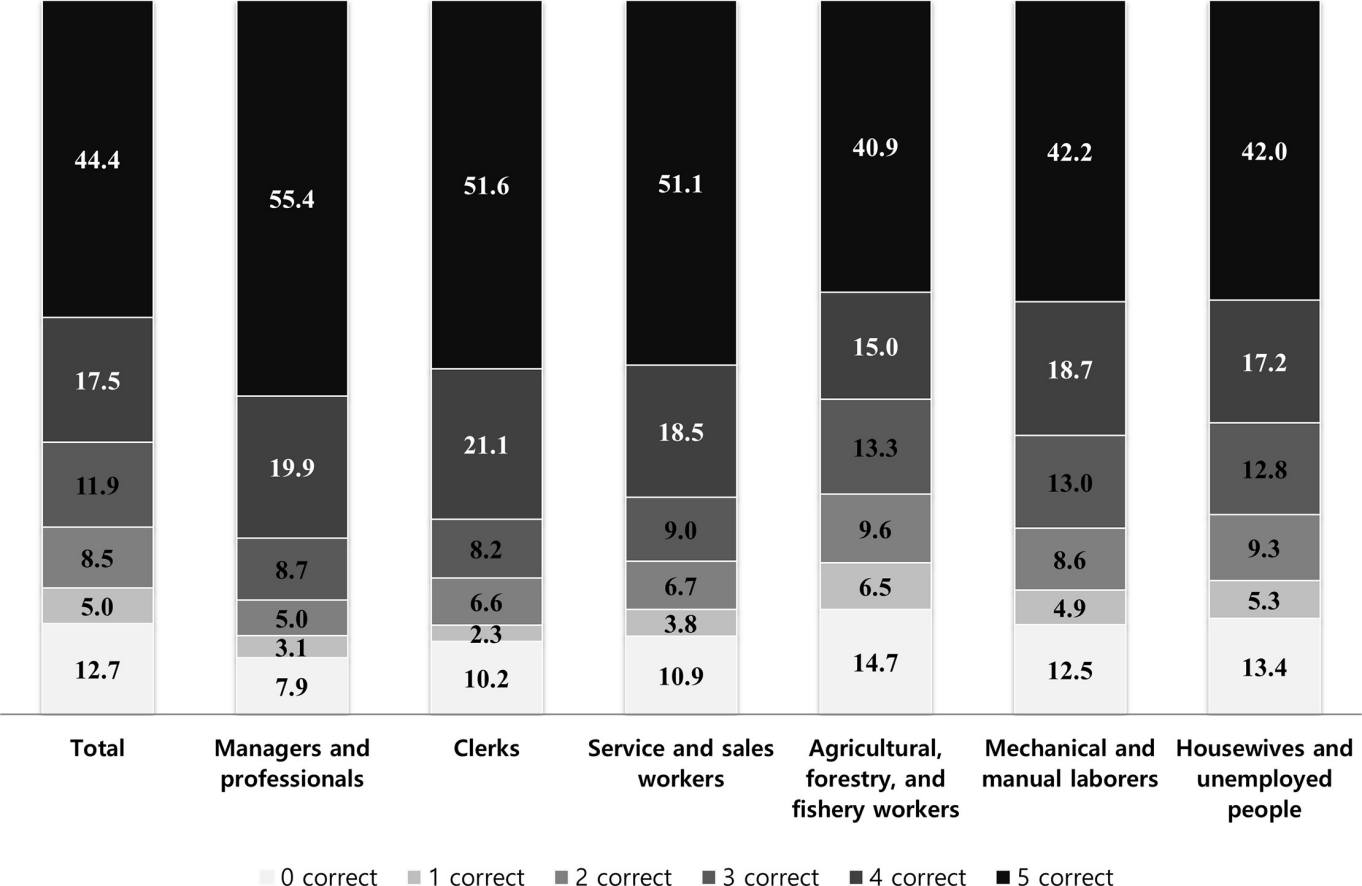
Proportions among occupations regarding knowledge of stroke warning symptoms. The proportions of those aware of stroke warning signs were 12.7%, 5.0%, 8.5%, 11.9%, 17.5%, and 44.4% with respect to knowing none, one, two, three, four, and five stroke warning signs, respectively.

The proportions of those aware of SWS were 12.7%, 5.0%, 8.5%, 11.9%, 17.5%, and 44.4% with respect to knowing none, one, two, three, four, and five SWS, respectively. The proportions of those aware of all five SWS were highest among MP (55.4%) and lowest among AFFW (40.9%). In contrast, the proportions of those aware of no stroke symptom were highest among AFFW (14.7%) and lowest among MP (7.9%) (Fig 2).

### Knowledge of SWS by occupation

Regarding S1 (sudden numbness or weakness), compared to MP (reference), the ORs for knowing S1 among AFFW, MML, and HUP were lower in Model 1 and Model 2, but the OR of SSW was lower only in Model 2. After fully adjusting for socio-demographic factors (Model 3), only AFFW had a significantly lower OR (0.74; 95% CI 0.59–0.92) for knowing S1 compared to MP. Regarding S2 (sudden difficulty speaking or understanding speech), compared to MP, the ORs for knowing S2 among SSW, AFFW, MML, and HUP were lower in all models. AFFW had the lowest OR (0.56; 95% CI 0.44–0.72) for knowing S2 than MP in Model 3. Regarding S3 (sudden dizziness), compared to MP, the ORs for knowing S3 among AFFW, MML, and HUP were lower in all models. Regarding S4 (sudden visual impairment), compared to MP, the ORs for knowing S3 among CL, SSW, AFFW, MML, and HUP were lower in all models. MML had the lowest OR (0.62; 95% CI 0.50–0.76) for knowing S3 than MP in Model 3. Regarding S5 (sudden severe headache), compared to MP, the ORs for knowing S5 among AFFW, MML, and HUP were lower in all models (Table 3).

**Table 3:** Odds ratios and 95% confidence intervals for occupational knowledge of stroke warning signs.

	Managers and professionals		Clerks		Service and sales workers		Agricultural, forestry, and fishery workers		Mechanical and manual laborers		Housewives and unemployed people	
Sudden numbness or weakness (S1)												
Model 1	1.00	(reference)	0.93	(0.73–1.20)	0.84	(0.68–1.04)	0.70	(0.58–0.85)	0.81	(0.66–0.99)	0.77	(0.64–0.93)
Model 2	1.00	(reference)	0.93	(0.72–1.20)	0.79	(0.64–0.98)	0.67	(0.55–0.82)	0.80	(0.65–0.98)	0.78	(0.64–0.94)
Model 3	1.00	(reference)	0.95	(0.74–1.22)	0.81	(0.65–1.01)	0.74	(0.59–0.92)	0.87	(0.70–1.08)	0.83	(0.68–1.03)
Sudden difficulty speaking or understanding speech (S2)												
Model 1	1.00	(reference)	0.84	(0.63–1.12)	0.66	(0.52–0.84)	0.42	(0.34–0.53)	0.57	(0.45–0.72)	0.48	(0.39–0.59)
Model 2	1.00	(reference)	0.82	(0.62–1.10)	0.66	(0.52–0.85)	0.47	(0.38–0.59)	0.59	(0.47–0.74)	0.57	(0.46–0.72)
Model 3	1.00	(reference)	0.85	(0.63–1.14)	0.68	(0.53–0.87)	0.56	(0.44–0.72)	0.67	(0.52–0.86)	0.64	(0.50–0.81)
Sudden dizziness (S3)												
Model 1	1.00	(reference)	0.92	(0.69–1.22)	0.83	(0.66–1.06)	0.49	(0.40–0.60)	0.55	(0.44–0.68)	0.55	(0.45–0.68)
Model 2	1.00	(reference)	0.90	(0.68–1.19)	0.86	(0.68–1.09)	0.57	(0.46–0.71)	0.58	(0.47–0.73)	0.70	(0.56–0.86)
Model 3	1.00	(reference)	0.91	(0.69–1.21)	0.85	(0.67–1.09)	0.63	(0.50–0.80)	0.63	(0.50–0.80)	0.74	(0.59–0.93)
Sudden visual impairment (S4)												
Model 1	1.00	(reference)	0.73	(0.58–0.93)	0.70	(0.57–0.85)	0.45	(0.38–0.54)	0.49	(0.40–0.59)	0.50	(0.42–0.59)
Model 2	1.00	(reference)	0.71	(0.56–0.91)	0.75	(0.61–0.92)	0.56	(0.46–0.68)	0.53	(0.44–0.65)	0.65	(0.54–0.79)
Model 3	1.00	(reference)	0.73	(0.57–0.92)	0.79	(0.64–0.98)	0.68	(0.55–0.84)	0.62	(0.50–0.76)	0.75	(0.62–0.92)
Sudden severe headache (S5)												
Model 1	1.00	(reference)	0.91	(0.72–1.15)	0.85	(0.70–1.04)	0.50	(0.42–0.60)	0.61	(0.51–0.74)	0.54	(0.46–0.64)
Model 2	1.00	(reference)	0.89	(0.70–1.14)	0.89	(0.72–1.09)	0.59	(0.49–0.71)	0.65	(0.54–0.79)	0.67	(0.56–0.81)
Model 3	1.00	(reference)	0.92	(0.73–1.18)	0.92	(0.74–1.14)	0.68	(0.56–0.84)	0.73	(0.60–0.90)	0.72	(0.59–0.88)
Knowing at least one of the stroke warning signs												
Model 1	1.00	(reference)	0.76	(0.53–1.10)	0.70	(0.51–0.96)	0.50	(0.38–0.66)	0.60	(0.45–0.81)	0.56	(0.42–0.73)
Model 2	1.00	(reference)	0.76	(0.52–1.09)	0.68	(0.50–0.94)	0.51	(0.38–0.69)	0.61	(0.45–0.83)	0.60	(0.45–0.80)
Model 3	1.00	(reference)	0.77	(0.53–1.11)	0.62	(0.45–0.86)	0.49	(0.36–0.67)	0.57	(0.42–0.79)	0.55	(0.40–0.75)
Knowing all five stroke warning signs												
Model 1	1.00	(reference)	0.86	(0.70–1.06)	0.84	(0.70–1.01)	0.56	(0.47–0.66)	0.59	(0.50–0.70)	0.58	(0.50–0.68)
Model 2	1.00	(reference)	0.84	(0.68–1.04)	0.87	(0.72–1.04)	0.64	(0.54–0.76)	0.62	(0.52–0.74)	0.71	(0.60–0.84)
Model 3	1.00	(reference)	0.87	(0.70–1.08)	0.95	(0.79–1.15)	0.79	(0.66–0.96)	0.76	(0.63–0.91)	0.84	(0.71–1.01)

Data are presented as odds ratio (95% confidence interval). Model 1: unadjusted. Model 2: adjusted for gender and age. Model 3: adjusted for gender, age, residence type, education level, marital status, and monthly household income.

### Association of occupation with awareness of SWS and emergency response

Compared to MP, the ORs for knowing at least one of the SWS among SSW, AFFW, MML, and HUP were lower in all models. After full adjustment (Model 3), AFFW (OR 0.49; 95% CI 0.36–0.67) had the lowest OR for knowing at least one of the SWS. Next, compared to MP, the ORs for knowing all five SWS among AFFW, MML, and HUP were lower in Models 1 and 2. After full adjustment (Model 3), the significance persisted among AFFW (OR 0.79; 95% CI 0.66–0.96) and MML (OR 0.76; 95% CI 0.63–0.91) (Table 3).

There was no significant association between occupation and correct emergency response (calling 119) in Model 1. After adjusting for gender and age (Model 2), the OR for correct emergency response among AFFW (OR 1.42; 95% CI 1.11–1.82) was higher. However, compared to MP, no occupation showed any significant difference in OR for correct emergency response after full adjustment (Model 3) (Table 4).

**Table 4:** Odds ratios and 95% confidence intervals for occupational knowledge of correct emergency response for stroke.

	Managers and professionals		Clerks		Service and sales workers		Agricultural, forestry, and fishery workers		Mechanical and manual laborers		Housewives and unemployed people	
Correct emergency response (calling 119 when someone shows stroke warning signs)												
Model 1	1.00	(reference)	1.09	(0.80–1.47)	1.09	(0.85–1.41)	1.14	(0.91–1.44)	0.99	(0.78–1.26)	0.84	(0.68–1.04)
Model 2	1.00	(reference)	1.06	(0.78–1.43)	1.14	(0.88–1.48)	1.42	(1.11–1.82)	1.07	(0.84–1.37)	1.16	(0.92–1.47)
Model 3	1.00	(reference)	1.05	(0.77–1.42)	1.15	(0.88–1.50)	1.30	(0.99–1.70)	1.12	(0.86–1.45)	1.18	(0.92–1.52)

Data are presented as odds ratio (95% confidence interval). Model 1: unadjusted. Model 2: adjusted for gender and age. Model 3: adjusted for gender, age, residence type, marital status, education level, and monthly household income.

## Discussion

Among the community-dwelling population, there was a significant difference in awareness of SWS by occupation. The awareness of at least one of the SWS was significantly lower among AFFW, MML, SSW, and HUP compared to MP. In addition, the awareness of all five SWS was significantly lower in AFFW and MML than MP. Meanwhile, no occupational difference was found in the awareness of correct emergency response for stroke.

Many stroke patients had little stroke knowledge, so they waited for their SWS to be relieved without seriously considering their SWS. Prospective studies pointed out that more than half of the prehospital delay in acute stroke was caused by the hesitation to contact emergency medical services (EMS) [24,25]. Many stroke patients are still unable to arrive at the hospital within the golden time due to lack of knowledge on SWS [14]. Stroke awareness had a significant impact on early arrival at the hospital [26–28], although some studies have found that previous stroke knowledge in patients was not associated with decision delay or EMS use [25,29]. Therefore, it should be emphasized to the general public not only to quickly recognize SWS by patients/bystanders, but also to contact the EMS immediately when stroke symptoms develop. Since bystanders play a critical role in the decision to contact the EMS [26], stroke public campaigns and education programs targeting the general population are important.

The risk of stroke differs by occupation. A recent nationwide cohort study conducted in Korea identified inequalities in cause-specific mortality, including stroke, depending on the occupation [23]. Age-standardized stroke mortality rate was highest for AFFW and lowest for professionals among males (rate ratio 2.76), while highest for elementary occupations and lowest for professionals among females (rate ratio 2.25) [23]. In a study of Japanese working-age males, compared to sales workers, those in the occupations of service, administrative and managerial, agriculture and fisheries, construction and mining, electricity and gas, transport, and professional/engineering had a higher risk of stroke mortality [22]. Manual workers and workers in the service industry had a higher risk of stroke occurrence than professionals/managers among middle-aged females [21]. Meta-analysis showed that employees who work long hours have a higher incidence of stroke than those working standard hours [30]. Disparities in cardiovascular health status were observed among U.S. occupational groups [31]. Framingham risk scores of technicians and elementary occupations were highest in Korea [32]. Taking into consideration the recent cohort study [23] and our study findings, stroke awareness in Korea was lowest among occupational groups with high stroke mortality. This suggests that, even if not all, low stroke awareness might contribute to stroke mortality. In addition, this shows that public health efforts are needed to improve awareness of vulnerable occupational groups with high stroke mortality and low stroke awareness.

A previous study of data from the 2013 KCHS showed a difference between occupation and pathway of awareness of the term cardiovascular-disease [33]. Compared to the reference group, AFFW and MML had lower awareness of the term cardiovascular-disease in various pathways (internet, television/radio/advertising on outdoor or subway, and hospitals/clinics). In Korea, rural areas where most AFFW live have fewer medical institutions than urban areas, while there are some public health institutions (public health centers, subcenters, and primary health care centers) in rural areas. In addition, MML had lower awareness of the term cardiovascular-disease in the pathway of public health institutions.

Korean farmers and fishermen have relatively low digital access to knowledge and information. The level of digital information by occupation was highest among MP/CL, students, and SSW and lowest in farmers/fishermen, production-related workers, and the unemployed [34]. In addition, the utilization rate of information and news search among farmers and fishermen (58.9%) was much lower than that of the general public (84.9%) [34]. For this reason, FFW and MML might have low digital access to stroke-related health information in Korea. Therefore, public health strategies for improving health literacy and reducing barriers to seeking stroke-related information should be developed. Assessment of health literacy among FFW and MML by health care professionals and education on how to access credible sources of stroke-related information with ease could improve the limited health literacy [35]. Community-based awareness campaign and education strategies can effectively improve public stroke awareness of the SWS and the need to call the EMS [36,37].

This study had several limitations. First, because this study utilized a cross-sectional design, it cannot infer a causal relationship. Second, this study assessed stroke knowledge using a closed-ended questionnaire. Closed-ended methods are more likely to generate a positive response when compared to open-ended methods, because they can provide immediate prompts for respondents to what the correct answer should be [13]. When using closed-ended questions to assess knowledge of SWS, the act of asking can lead to different types of responses by allowing respondents to indicate what their answers could be. This question technique can reflect not only a knowledge of SWS but also hidden beliefs about SWS recognition. Third, respondents were given only five positive questions (identifying correct SWS) without negative or trap questions (identifying incorrect SWS). This approach may falsely increase the knowledge of SWS, hence awareness of SWS of the respondents might be overestimated. Fourth, the occupation of the respondents was classified by identifying the type of work currently engaged in. However, occupation variables did not include more specific features such as duration of employment, turnover, retirement timing, and intensity of work. Despite these limitations, this study is valuable as the first study to fully evaluate the association between occupation and stroke awareness in Korea. We also evaluated data from representative samples of the general population, including a large number of community-dwelling adults without stroke.

## Conclusions

We identified differences of knowledge of SWS by occupation in a community-dwelling population. AFFW and MML have the lowest awareness of SWS. To improve stroke literacy and to reduce the disparity of awareness of SWS in community settings, public health efforts including campaign and education with an emphasis on AFFW and MML are needed.
